# Sodium-Ion-Free Fermentative Production of GABA with *Levilactobacillus brevis* CD0817

**DOI:** 10.3390/metabo13050608

**Published:** 2023-04-28

**Authors:** Haixing Li, Jinfeng Pei, Cheng Wei, Zhiyu Lin, Hao Pan, Zhenkang Pan, Xinyue Guo, Zhou Yu

**Affiliations:** 1 State Key Laboratory of Food Science and Technology, Nanchang University, Nanchang 330047, China; hxli@ncu.edu.cn (H.L.); 412345420117@email.ncu.edu.cn (J.P.); 412345420018@email.ncu.edu.cn (C.W.); 402531320008@email.ncu.edu.cn (Z.L.); 405800210015@email.ncu.edu.cn (H.P.); pzk2894866116@163.com (Z.P.); gxy982238296@163.com (X.G.); 2 Sino-German Joint Research Institute, Nanchang University, Nanchang 330047, China; 3 School of Chemistry and Chemical Engineering, Nanchang University, Nanchang 330031, China

**Keywords:** gamma-aminobutyric acid, sodium-ion-free GABA fermentation, *Levilactobacillus brevis* CD0817, L-glutamic acid, culture condition optimization

## Abstract

Gamma-aminobutyric acid (GABA) has positive effects on many physiological processes. Lactic acid bacterial production of GABA is a future trend. This study aimed to produce a sodium-ion-free GABA fermentation process for *Levilactobacillus brevis* CD0817. In this fermentation, both the seed and fermentation media used L-glutamic acid instead of monosodium L-glutamate as the substrate. We optimized the key factors influencing GABA formation, adopting Erlenmeyer flask fermentation. The optimized values of the key factors of glucose, yeast extract, Tween 80, manganese ion, and fermentation temperature were 10 g/L, 35 g/L, 1.5 g/L, 0.2 mM, and 30 °C, respectively. Based on the optimized data, a sodium-ion-free GABA fermentation process was developed using a 10-L fermenter. During the fermentation, L-glutamic acid powder was continuously dissolved to supply substrate and to provide the acidic environment essential for GABA synthesis. The current bioprocess accumulated GABA at up to 331 ± 8.3 g/L after 48 h. The productivity of GABA was 6.9 g/L/h and the molar conversion rate of the substrate was 98.1%. These findings demonstrate that the proposed method is promising in the fermentative preparation of GABA by lactic acid bacteria.

## 1. Introduction

Gamma-aminobutyric acid (GABA) represents a natural amino acid that widely exists in numerous organisms [1,2]. In mammals, GABA mainly acts as an inhibitory neurotransmitter [3,4], while also being responsible for many other physiological functions. For instance, GABA has therapeutic effects in liver injury [5], hepatic encephalopathy [6], and cardiovascular diseases [7]. Hence, GABA has already been identified as a functional factor that can be applied to the food and pharmaceutical areas. As a rising star metabolite, GABA has garnered a significant amount of attention for decades [8].

Over the past three decades, the use of lactic acid bacteria to synthesize GABA has been extensively investigated because of the safety of this species [9,10,11]. Consequently, the isolation a GABA-producing lactic acid bacterium is an essential first step. The prevailing isolation methods are based on analysis of the GABA end product [12,13,14]. Many target strains have been isolated using these methods [15,16,17,18]. However, DNA-sequence-based methods are attractive methods in this field [19,20,21,22,23,24,25,26,27].

Some GABA-producing lactic acid bacterial strains have displayed potential for industrial application. Investigators have been striving to devise practical strategies for enhancing their GABA production efficiency. As a modern cutting-edge technology, genetic engineering could effectively improve the GABA production of some lactic acid bacteria [28,29,30]. However, a traditional scheme remains a useful avenue for enhancing GABA production. In fact, a traditional scheme possesses some incomparable advantages, characterized by the process of stability, safety, and convenience, and it will continue to play an irreplaceable role in optimizing fermentation in the foreseeable future [31,32,33,34,35].

Most of the available GABA fermentations use monosodium L-glutamate as the substrate. Recently, our lab designed a pH auto-sustain-based fermentation strategy, using L-glutamic acid as a substrate, for the efficient production of GABA by *L. brevis* CD0817 [36]. However, this fermentation uses an inoculum containing 28 g/L of monosodium L-glutamate, which still brings an appreciable amount of Na^+^ into the fermentation broth [36,37]. On the one hand, the sodium ion (Na^+^) introduced by monosodium L-glutamate suppresses microbial growth and metabolism and, on the other hand, it reduces the quality of the fermentative product [38]. Therefore, it would be useful to construct an Na^+^-free GABA fermentation bioprocess for a lactic acid bacterial strain that may overcome the issues arising from Na^+^.

In this study, we reported an Na^+^-free bioprocess for *L. brevis* CD0817 to synthesize GABA. To achieve this, we used seed and fermentation media without Na^+^. We first optimized the pivotal fermentative parameters using Erlenmeyer flask experiments, and then set up the Na^+^-free bioprocess in a 10-L fermenter. The results indicated that this new bioprocess exhibits more efficient GABA synthesis than the previous pH auto-sustain-based fermentation method.

## 2. Materials and Methods

### 2.1. Reagents

The yeast extract used was a product of the Angel Yeast Co., Ltd. (Wuhan, China). L-glutamic acid was purchased from the Dragon Biotech Co., Ltd. (Emeishan, China). Monosodium L-glutamate was produced by the Lanji Technology Development Co., Ltd. (Shanghai, China). In addition, the reagent of 3,5-dinitrosalicylic acid was provided by the Solarbio Science and Technology Co., Ltd. (Beijing, China).

The derivatization reagent for amino acids was manufactured by dissolving o-phthalaldehyde (10 mg) and β-mercaptoethanol (10 μL) in acetonitrile (2.5 mL). The borate buffer was produced as follows: 4.9 g boric acid was weighed and added to 100 mL of double-distilled water; the pH was adjusted to 10.4 with 1 M NaOH solution; and finally, it was diluted to 200 mL with double-distilled water. The mobile phase used in HPLC was prepared as follows: triethylamine (200 μL) and sodium acetate trihydrate (2.7 g) were added to double-distilled water (0.9 L); the pH was adjusted to 7.3 with glacial acetic acid; then, the mixture was made up to 1 L with double-distilled water, and finally was supplemented with 250 mL acetonitrile [39].

### 2.2. Strain, Media, and Preparation of the Inoculum

*L. brevis* CD0817 was the lactic acid bacterial strain used in this work. The strain was originated from a healthy adult fecal sample [16]. *L. brevis* CD0817 showed a GABA production of 321 g/L after 48 h of pH auto-sustain-based fermentation [36]. 

The GYM medium included a 35 g/L yeast extract, 0.3 mM (50 mg/L) MnSO_4_·H_2_O, 28 g/L monosodium L-glutamate, 1 g/L Tween 80, and 10 g/L glucose. The seed medium, namely the Na^+^-free seed medium, included 35 g/L yeast extract, 0.3 mM MnSO_4_·H_2_O, 150 g/L of L-glutamic acid, 1 g/L Tween 80, and 10 g/L glucose. The starting fermentation medium contained 35 g/L yeast extract, 0.3 mM MnSO_4_·H_2_O, 1 g/L Tween 80, and 5 g/L glucose; 650 g/L sterile L-glutamic acid powder was added when initiating fermentation. L-glutamic acid, glucose, and the remaining nutrients were separately autoclaved under 121 °C for 20 min and mixed just before use [36,40].

The inoculum of *L. brevis* CD0817 was produced as follows: the cells were streaked onto a GYM agar plate and subsequently cultured under 30 °C for 2 d. A bacterial colony was isolated to a tube containing 5 mL GYM medium, followed by culturing at 30 °C for 1 d. The cultured broth was inoculated to an Erlenmeyer flask (250 mL) containing 100 mL of the seed medium, then cultured at 30 °C and 100 rpm until its *A*_600_ reached approximately 3.0–3.5.

### 2.3. Optimization Trials

The Na^+^-free inoculum, recipe for the fermentative medium (glucose, yeast extract, manganese ions, and Tween 80), and culture temperature were successively optimized in this work, so as to maximize the GABA synthesis efficiency of *L. brevis* CD0817. Please note, once a factor was optimized, its optimization value was adopted to improve the fermentation medium, while the other components remained unchanged. Then, the next optimization was carried out in this improved medium. In accordance with this goal, fermentations were performed using a 250-mL Erlenmeyer flask. Each fermentation was primed by inoculating a seed broth (*A*_600_ = 3.0–3.1) into a flask loaded with 100 mL of the fermentative medium and 65 g L-glutamic acid powder. The inoculum size was 10% (*v*/*v*). The fermentations were carried out statically at 30 °C for 72 h. Samples were drawn every 12 h and were stored at −20 °C prior to assay. Each fermentation experiment was repeated three times. Unless otherwise emphasized, the above experimental conditions were universal for all the optimizations. The other details for each optimization are shown below.

#### 2.3.1. Effects of Na^+^-Free Inoculum

To assess the effects of the Na^+^-free inoculum on GABA fermentation, at the time points when the *A*_600_ value of a seed culture reached 1, 1.5, 2, 2.5, 3, or 4, it was inoculated to initiate the corresponding fermentation. The fermentation medium used was the starting fermentation medium.

#### 2.3.2. Effects of Glucose

To assess the effects of glucose on GABA fermentation, a seed culture was inoculated to the fermentation media containing various levels (0, 2.5, 5, 7.5, 10, 20, and 40 g/L) of glucose to initiate fermentation. The other components in the fermentation media were identical to those in the starting fermentation medium.

#### 2.3.3. Effects of Yeast Extract

To assess the effects of yeast extract on GABA fermentation, a seed culture was inoculated to the fermentation media containing various levels (0, 15, 25, 30, 35, 40, and 50 g/L) of yeast extract to initiate fermentation. The other components in the fermentation media were 10 g/L glucose, 0.3 mM MnSO_4_·H_2_O, and 1 g/L Tween 80.

#### 2.3.4. Effects of Manganese Ions

To assess the effects of manganese ions on GABA fermentation, a seed culture was inoculated to the fermentation media containing various levels (0, 0.2, 0.25, 0.3, 0.35, 0.4, and 0.6 mM) of manganese ions to initiate fermentation. The other components in the fermentation media were 10 g/L glucose, 35 g/L yeast extract, and 1 g/L Tween 80.

#### 2.3.5. Effects of Tween 80

To assess the effects of Tween 80 on GABA fermentation, a seed culture was inoculated to the fermentation media containing various levels (0, 0.5, 1, 1.5, 2, 2.5, and 3 g/L) of Tween 80 to initiate fermentation. The other components in the fermentation media were 10 g/L glucose, 35 g/L yeast extract, and 0.2 mM MnSO_4_·H_2_O.

#### 2.3.6. Effects of Temperature

To assess the effects of temperature on GABA fermentation, a seed culture was inoculated to the optimized fermentation media to initiate fermentation. Fermentations were performed at different temperatures (25, 30, 35, 40, and 45 °C), respectively. The optimized fermentation medium was composed of 35 g/L yeast extract, 10 g/L glucose, and 0.2 mM MnSO_4_·H_2_O, plus 1.5 g/L Tween 80.

### 2.4. Process of Substrate Consumption

A total of 100 mL of optimized fermentation medium was poured into a 250 mL flask containing 65 g L-glutamic acid powder just prior to fermentation. The fermentation was initiated by inoculating 10% seed broth; then, it was statically fermented at 30 °C for 48 h. The fermentation was recorded and photographed every 12 h.

### 2.5. Fermenter Trials

The inoculum was prepared as previously mentioned. The fermentation was initiated by transferring the inoculum (*A*_600_ = 3.0–3.1) at an amount of 10% (*v*/*v*) into a 10-L fermenter loaded with 4 L of the optimized medium and 2600 g L-glutamic acid powder. The fermentation was hermetically conducted under a constant agitation of 50 rpm under 30 °C for 48 h. The cover of the fermenter was connected to a silicone pipe for exhausting carbon dioxide. Another end of the pipe was immersed in water to isolate air. Samples were aseptically taken at the interval of 2 h and stored at −20 °C for their next use. The fermenter trial was carried out in triplicate. 

### 2.6. Analytical Methods

To improve analysis efficiency, we adopted the SINICS (sensitivity intensified ninhydrin-based chromogenic system) method to analyze the flask samples. This method depends on the SINICS reagent. A 2.9 mL aliquot of this reagent was constituted by 1% (*w*/*v*) ninhydrin, 35 μL 0.2 M sodium acetate buffer (pH 5), 40% (*v*/*v*) ethanol, and 25% (*v*/*v*) ethyl acetate. The SINICS assay was operated as follows: a 0.1 mL 400-fold-diluted sample was mixed with the SINICS reagent (2.9 mL), and it was incubated under 70 °C for 30 min; then, the reading was obtained using an UV1200B spectrophotometer (Mapada Instruments, Shanghai, China). The GABA content was expressed as a net *A*_570_ value (absorbance at 570 nm), subtracting the background control. In this study, the background control was a saturated aqueous solution of L-glutamic acid at 30 °C. Please note, a sample without visible L-glutamic acid powder taken from the anaphase of fermentation should be saturated with L-glutamic acid powder preceding SINICS analysis, so as to ensure it has a comparable substrate level to the background control [41].

The GABA level of the fermenter sample was analyzed using the HPLC method. The pre-treatment, derivatization, and determination of the sample were carried out according to the method described by Chen et al. [39]. 

The cell biomass was represented as an *A*_600_ value. The experimental process was as follows: the fermentation broth was shaken well, followed by standing for 1–2 min to allow the majority of the L-glutamic acid powder to settle; then, a sample was drawn from the upper portion of the fermentation broth. The sample underwent 5 min of centrifugation at 5000× *g*; and the cell pellet that contained a little L-glutamic acid powder was recovered. The pellet was resuspended in a 0.8% NaCl solution to completely dissolve the L-glutamic acid powder. The cells were recovered by 5 min of centrifugation at 5000× *g*; they were resuspended in 0.8% NaCl solution; and finally, the *A*_600_ was checked using the spectrophotometer. 

Residual glucose was assessed with the reagent 3,5-dinitrosalicylic acid. In brief, 2 mL of the reagent was added to a 1-mL 10-fold-diluted sample; this was boiled for 5 min, quickly cooled with tap water, and supplemented with 9 mL of distilled water; then, the *A*_540_ was obtained using the spectrophotometer.

### 2.7. Calculations

The formula provided below was adopted to obtain the molar conversion rate (*k*) of the substrate L-glutamic acid:

(1) $$k=\frac{147.1C_{GABA}V}{103.1m_{Glu}}\times 100\%$$

where *C_GABA_* represents the GABA concentration (g/L), *V* represents the volume (L) of the fermentation broth, *m_Glu_* represents the mass (g) of consumed L-glutamic acid, 147.1 represents the molar mass (g/mol) of L-glutamic acid, and 103.1 represents the molar mass (g/mol) of GABA.

### 2.8. Statistical Analysis

We plotted all the figures with the Origin 2022 software (OriginLab, Northampton, MA, USA). The data were presented as means ± standard deviations.

## 3. Results and Discussion

In our former study, glucose, yeast extract, Tween 80, Mn^2+^, and temperature were determined as the vital parameters modulating the synthesis of GABA by *L. brevis* CD0817 [36]. This study aimed to specify the levels of these factors to adapt the current Na^+^-free fermentation.

### 3.1. Effects of Na^+^-Free Inoculum

**Figure 1:**
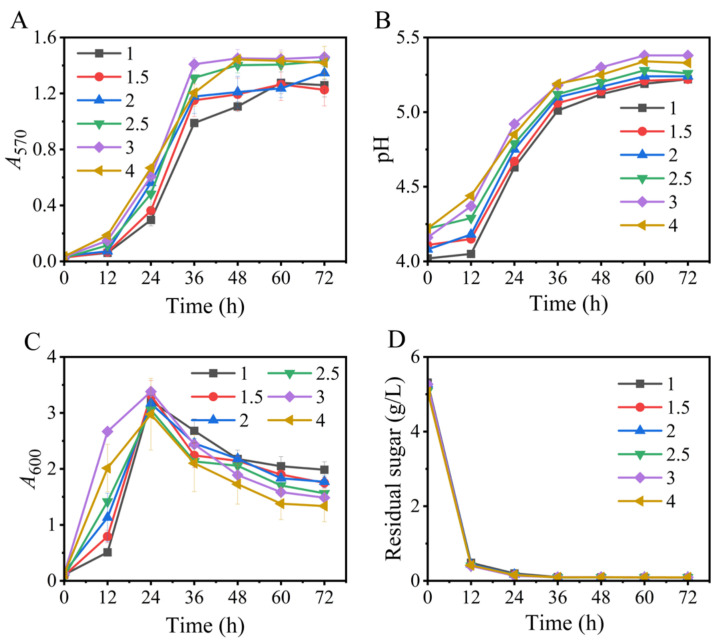
Effects of the *A*_600_ value of sodium-ion-free inoculum on GABA synthesis (**A**), pH (**B**), cell growth (**C**), and glucose consumption (**D**) in fermentation. *A*_570_ and *A*_600_ represent GABA content and cell growth, respectively. Vertical bars depict standard deviations of means. GABA represents gamma-aminobutyric acid; *A*_570_ and *A*_600_ indicate absorbance at 570 and 600 nm, respectively.

We first tested the effects of the Na^+^-free inoculum on GABA synthesis. The results illustrated that all the fermentations, respectively initiated by inocula with various *A_600_* values (1, 1.5, 2, 2.5, 3, and 4), could efficiently produce GABA. These data suggested that it was feasible to use an Na^+^-free medium to produce the seed culture. Moreover, changes in the *A*_600_ value of the inoculum from 1 to 4 had little impact on the fermentation indicators, including GABA synthesis, pH variation, cell growth, and glucose consumption (Figure 1), implying a flexibility in inoculation. Certainly, an inoculum with an *A*_600_ value of 2.5–4.0 was slightly improved (Figure 1). Next, we examined an inoculum with an *A*_600_ value between 3.0 and 3.5.

### 3.2. Effects of Glucose

**Figure 2:**
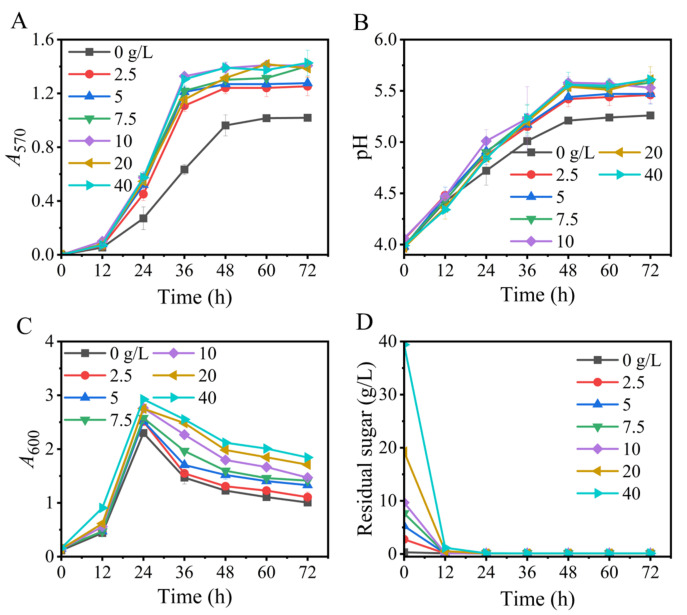
Effects of the level of glucose on GABA synthesis (**A**), pH (**B**), cell growth (**C**), and glucose consumption (**D**) in fermentation. *A*_570_ and *A*_600_ represent GABA content and cell growth, respectively. Vertical bars depict standard deviations of means. GABA represents gamma-aminobutyric acid; *A*_570_ and *A*_600_ indicate absorbance at 570 and 600 nm, respectively.

Glucose is a suitable carbon source for *L. brevis* strains to produce GABA [38,42]. In this section, we optimized the glucose level so as to maximize GABA generation in this new fermentation. The results illustrated that, on the one hand, the glucose level exerted a limited effect on the trend in GABA formation, which was similar across the tested glucose levels. Specifically, GABA was slowly synthesized from 0 to 12 h, rapidly synthesized from 12 to 36 h, and then peaked. On the other hand, the GABA titer varied with glucose concentration. In particular, the GABA titer in the no-glucose medium was much lower than that in the glucose-containing media. A 10- or 40-g/L concentration of glucose produced the highest GABA titer, and the other concentrations (10–40 g/L) of glucose produced slightly lower titers (Figure 2). 

GABA synthesis is positively correlated with cell number. Furthermore, the most suitable pH for the lactic acid bacterial production of GABA generally lies around 5, while an excessively high or low pH inhibits its production [2,43,44]. As a key energy source and structural substance, glucose can facilitate GABA production by promoting cell growth. Conversely, glucose also weakens GABA formation by converting itself to small molecular organic acids (mainly lactic acid), which further acidifies the low-pH environment caused by L-glutamic acid. Hence, the role of glucose depends on the comprehensive effect of its metabolism [36,37,40], rationalizing that 10 or 40 g/L of glucose favored GABA production. Here, 10 g/L glucose was used in the subsequent experiments.

### 3.3. Effects of Yeast Extract

**Figure 3:**
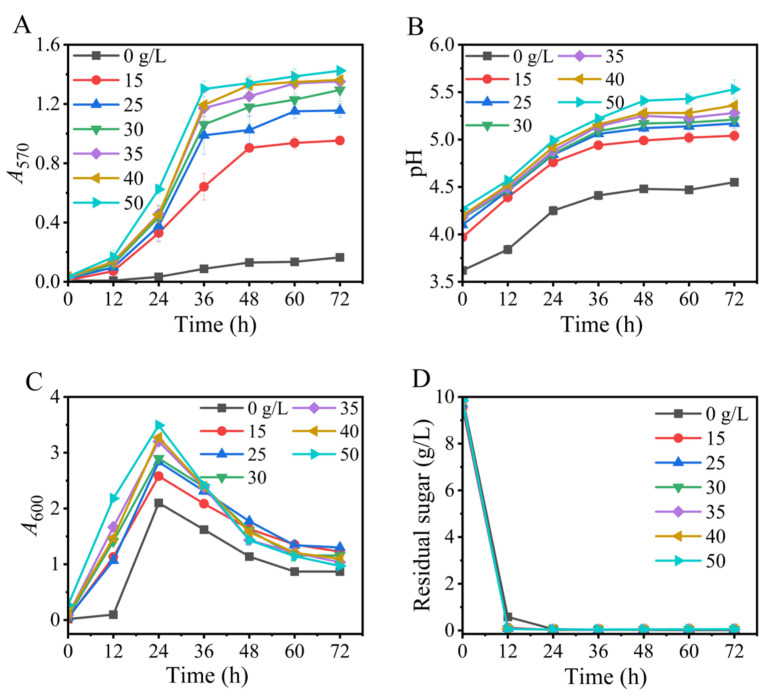
Effects of level of yeast extract on GABA synthesis (**A**), pH (**B**), cell growth (**C**), and glucose consumption (**D**) in fermentation. *A*_570_ and *A*_600_ represent GABA content and cell growth, respectively. Vertical bars depict standard deviations of means. GABA represents gamma-aminobutyric acid; *A*_570_ and *A*_600_ indicate absorbance at 570 and 600 nm, respectively.

The impact of yeast extract on GABA synthesis was next evaluated. The data illustrated that GABA production increased with an increase in nitrogen from 0 to 35 g/L. A higher level of nitrogen promoted cell growth but could not further benefit GABA formation (Figure 3). Therefore, 35 g/L of yeast extract was recommended. Nitrogen affects lactic acid bacterial GABA production in a strain-specific manner. For example, yeast extract, tryptone, and whey powder effectively support the GABA production of *L. plantarum* EJ2014 [45], *L. brevis* HYE1 [46], and *L. brevis* A3 [47], respectively.

### 3.4. Effects of Manganese Ions

**Figure 4:**
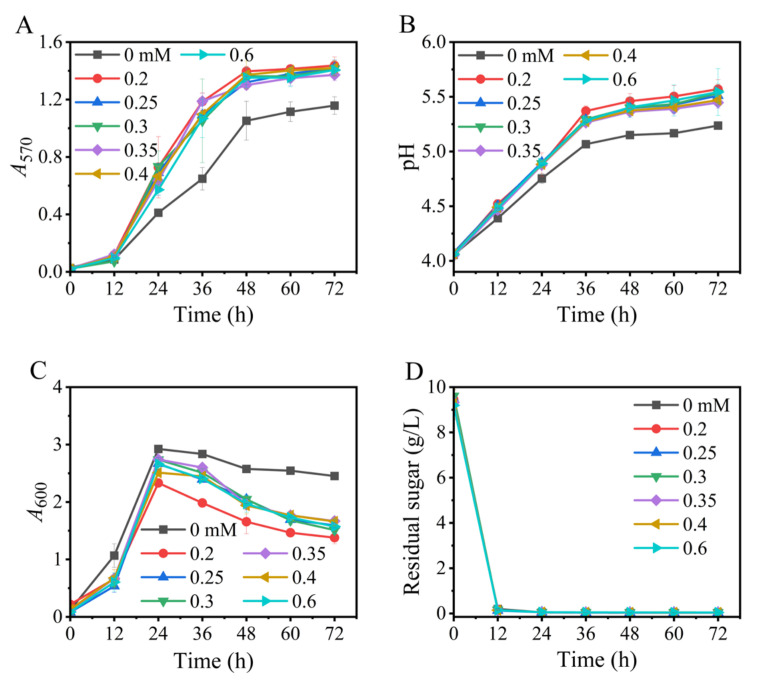
Effects of level of manganese ions on GABA synthesis (**A**), pH (**B**), cell growth (**C**), and glucose consumption (**D**) in fermentation. *A*_570_ and *A*_600_ represent GABA content and cell growth, respectively. Vertical bars depict standard deviations of means. GABA represents gamma-aminobutyric acid; *A*_570_ and *A*_600_ indicate absorbance at 570 and 600 nm, respectively.

We have verified that manganese ions (Mn^2+^) exhibited a significant effect on the existing GABA bioprocess of *L. brevis* CD0817 [36]. Herein, the potential role of Mn^2+^ in this Na^+^-free fermentation was evaluated. As is summarized in Figure 4, Mn^2+^ was also indispensable in the current process, and 0.2 mM of Mn^2+^ endorsed efficient GABA production. A higher level of Mn^2+^ (0.25–0.6 mM) could not further increase GABA formation, despite facilitating cell growth (Figure 4C). The underlying mechanism is currently unclear and deserves further study. 

### 3.5. Effects of Tween 80

**Figure 5:**
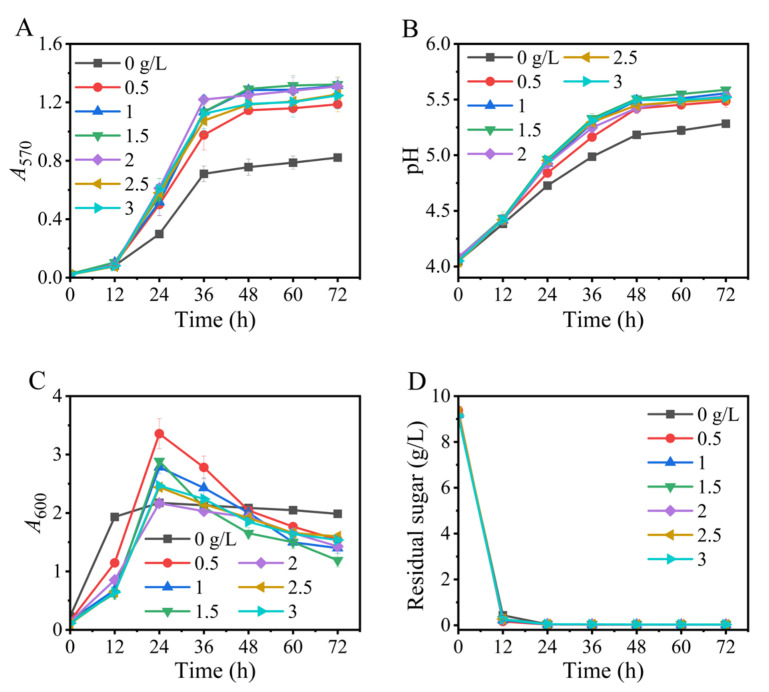
Effects of level of Tween 80 on GABA synthesis (**A**), pH (**B**), cell growth (**C**), and glucose consumption (**D**) in fermentation. *A*_570_ and *A*_600_ represent GABA content and cell growth, respectively. Vertical bars depict standard deviations of means. GABA represents gamma-aminobutyric acid; *A*_570_ and *A*_600_ indicate absorbance at 570 and 600 nm, respectively.

Tween 80 is a stimulator for lactic acid bacteria to produce biologic agents, including GABA [42], bacteriocins [48], and riboflavin [49]. Thus, the influences of the level of Tween 80 on GABA synthesis were examined here. As is shown in Figure 5A, GABA generation was gradually fortified as Tween 80 was increased from 0 to 1.5 g/L, resembling the previous findings [37,50,51]. However, a higher content of Tween 80 could not further boost GABA synthesis. Therefore, Tween 80 at a concentration of 1.5 g/L was selected to be used in this study.

### 3.6. Effects of Temperature

**Figure 6:**
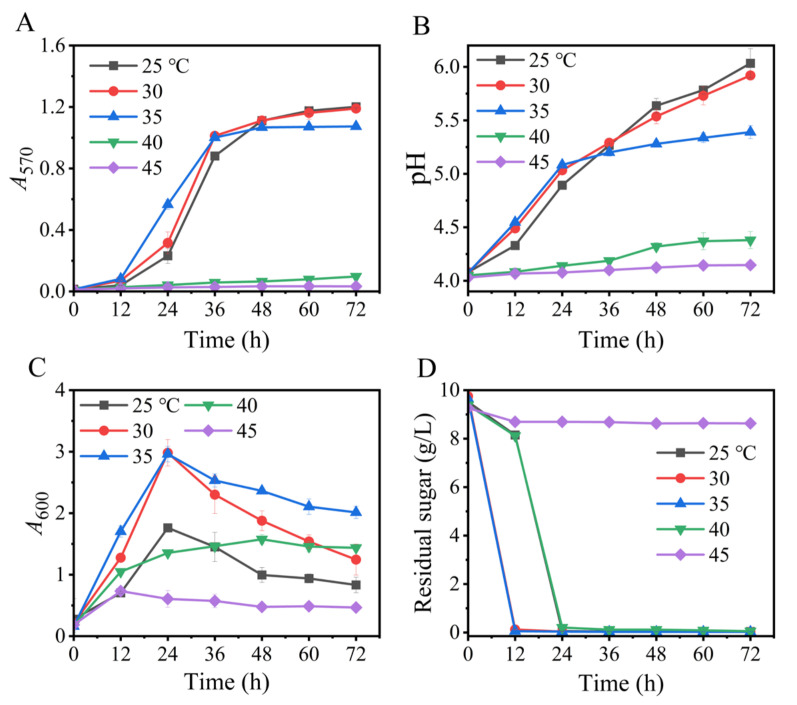
Effects of temperature on GABA synthesis (**A**), pH (**B**), cell growth (**C**), and glucose consumption (**D**) in fermentation. *A*_570_ and *A*_600_ represent GABA content and cell growth, respectively. Vertical bars depict standard deviations of means. GABA represents gamma-aminobutyric acid; *A*_570_ and *A*_600_ indicate absorbance at 570 and 600 nm, respectively.

In this section, we assessed the impact of temperature (25–45 °C) on the Na^+^-free GABA synthesis. As is shown, temperature significantly modulated GABA production (Figure 6), and a temperature of 25–30 °C maximized the synthesis of GABA. Although 35 °C was the most suitable temperature for the cell growth of *L. brevis* CD0817, this advantage did not translate to GABA synthesis. This is possibly because GAD is less stable at this temperature [36,52]. Overall, 25–30 °C was the appropriate temperature range for GABA production by this strain, similar to the findings of the pH auto-sustain-based fermentation and GABA fermentations of other *L. brevis* strains [37,42,53].

### 3.7. Process of Substrate Consumption

**Figure 7:**
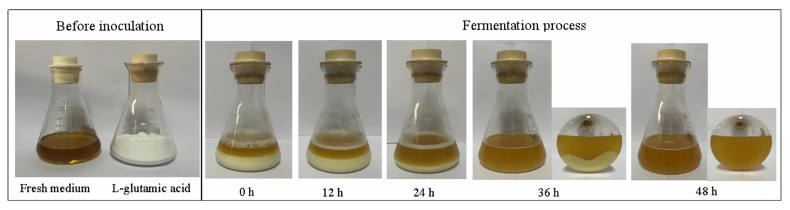
L-glutamic acid consumption during fermentation. The volume of fermentation broth was increased to approximately 136 mL when L-glutamic acid was completely converted.

Figure 7 illustrates the substrate consumption during the Na^+^-free GABA fermentation. The L-glutamic acid powder was gradually reduced as fermentation progressed, and depleted after 48 h, clearly depicting a sustained release of the substrate. This sustained release is crucial for L-glutamic-acid-based GABA bioconversion, because it substantially overcomes the inhibitory effects of the substrate on cell growth and metabolism [36,37,40].

### 3.8. Na^+^-Free GABA Bioprocess 

**Figure 8:**
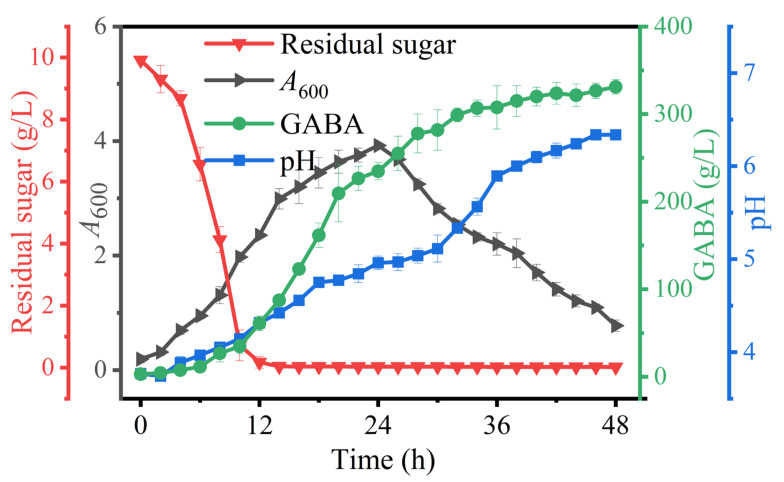
Sodium-ion-free GABA fermentation process implemented by *Levilactobacillus brevis* CD0817. *A*_600_ (absorbance at 600 nm) represents cell growth. GABA represents gamma-aminobutyric acid. Vertical bars depict standard deviations of means.

Based on the above findings, an Na^+^-free GABA fermentation process was tailored for *L. brevis* CD0817. The above findings also indicated that GABA was efficiently generated from 12 to 36 h (Figure 1, Figure 2, Figure 3, Figure 4, Figure 5, Figure 6 and Figure 7). Therefore, it is suggested that the Na^+^-free fermentation process should be carried out for 48 h. The Na^+^-free bioprocess was executed in the 10 L fermenter at 30 °C and 50 rpm. The fermenter was loaded with 4 L of the optimized medium, 10% (*v*/*v*) seed culture, and 2600 g solid L-glutamic acid. As is indicated in Figure 8, the cells rapidly grew immediately after inoculation, then declined after 24 h. The high osmolarity attributed to the high concentration of the product may contribute to this decline [36,54,55]. Glucose was almost depleted at 12 h, but the cells continued to grow until 24 h, possibly due to the carbon supply from the yeast extract [36]. Little GABA was accumulated during the first 6 h; then, it rapidly accumulated until approximately 36 h, and plateaued afterwards. The final GABA titer reached 331 ± 8.3 g/L. The productivity of GABA was 6.9 g/L/h, and the substrate conversion rate was 98.1%. Compared with the pH auto-sustain-based process [36], GABA production during this Na^+^-free fermentation was increased by 2.8%.

In a lactic acid bacterial cell, the glutamic acid decarboxylase (GAD) system, constituted by the L-glutamate/GABA antiporter and the GAD enzyme, is the mechanism for producing GABA. The synthesis of GABA involves three steps: first, L-glutamate is pumped into the cell by the antiporter; then, the L-glutamate is decarboxylated into GABA by the intra-cellular GAD; and finally, the GABA is pumped out of the cell by the anti-porter. Each decarboxylation reaction removes one H^+^ [56,57,58,59]. Hence, an acidic pH is indispensable for activating the GAD system and, in general, the optimal pH is approximately 5 [2]. In this study, the fermentative medium saturated with L-glutamic acid showed a rather low pH (3.5), which was elevated to 3.7 after the inoculation. The pH was gradually increased to approximately 6.3 after 48 h of fermentation. This means it is impossible to sustain the optimal pH across the fermentation. However, we have proven that a GAD-induced cell of *L. brevis* CD0817 can work even in an alkaline environment. This Na^+^-free fermentation should have provided a suboptimal acidity (pH 3.7–6.3) for the decarboxylation, which was verified by the high GABA production of 331 g/L (Figure 8).

Previously, monosodium L-glutamate was used as the substrate in GABA fermentation. Monosodium L-glutamate shows a neutral isoelectric point arising from an Na^+^ on the α-carboxyl group. In the case of using monosodium L-glutamate as a substrate, GABA formation is gradually weakened as fermentation progresses, because each decarboxylation contributes to the alkalization of the environment by consuming an H^+^. In order to maintain an acidic environment, an exogenous acid agent thus has to be continuously fed [29]. The introduced acid radical, however, suppresses GABA synthesis and simultaneously lowers the fermentative product quality [43,44,60]. 

More recently, our lab devised a pH auto-sustain-based scheme, using L-glutamic acid as the substrate, for the strain *L. brevis* CD0817 to efficiently ferment GABA. L-glutamic acid has at least two merits over monosodium L-glutamate: its low solubility and isoelectric point. First of all, even if all of the required substrate is added at once before fermentation, it would have little negative effect, because it mainly exists in the form of a powder. Second, with the progress of fermentation, the L-glutamic acid powder slowly dissolves to maintain the acidity required for the decarboxylation. Nevertheless, the seed medium used in the pH auto-sustain-based fermentation was still supplemented with 28 g/L monosodium L-glutamate. As a result, the inoculation undoubtedly introduced some Na^+^ into the fermentation broth [36]. Consequently, in the current study, we used L-glutamic acid instead of monosodium L-glutamate in the seed medium. The results demonstrated that the Na^+^-free seed culture did not bring about any negative effects on GABA fermentation.

## 4. Conclusions

In this study, an Na^+^-free GABA fermentation was constructed for *L. brevis* CD0817 to produce GABA. This was characterized by using L-glutamic acid as the substrate in both the seed and fermentation media. After 48 h of cultivation, the production and productivity of GABA reached 331 g/L and 6.9 g/L/h, respectively. Additionally, the substrate conversion rate reached 98.1%. This Na^+^-free strategy may be promoted for use with other lactic acid bacterial strains.

## Data Availability

The data presented in this study are available in this article.

## References

[1] Ueno H. (2000). Enzymatic and structural aspects on glutamate decarboxylase. J. Mol. Catal. B Enzym..

[2] Li H., Cao Y. (2010). Lactic acid bacterial cell factories for gamma-aminobutyric acid. Amino Acids.

[3] Yang Y., Rui Q., Han S., Wu X., Wang X., Wu P., Shen Y., Dai H., Xue Q., Li Y. (2022). Reduced GABA levels in the medial prefrontal cortex are associated with cognitive impairment in patients with NMOSD. Mult. Scler. Relat. Disord..

[4] Ting Wong C.G., Bottiglieri T., Snead O.C. (2003). GABA, γ-hydroxybutyric acid, and neurological disease. Ann. Neurol..

[5] Hata T., Rehman F., Hori T., Nguyen J.H. (2019). GABA, γ-aminobutyric acid, protects against severe liver injury. J. Surg. Res..

[6] Cohen B.I. (2002). The significance of ammonia/gamma-aminobutyric acid (GABA) ratio for normality and liver disorders. Med. Hypotheses.

[7] Han S.M., Lee J.S. (2017). Production and its anti-hyperglycemic effects of γ-aminobutyric acid from the wild yeast strain *Pichia silvicola* UL6-1 and *Sporobolomyces carnicolor* 402-JB-1. Mycobiology.

[8] Diana M., Quílez J., Rafecas M. (2014). Gamma-aminobutyric acid as a bioactive compound in foods: A review. J. Funct. Foods.

[9] Wu Q., Shah N.P. (2017). High γ-aminobutyric acid production from lactic acid bacteria: Emphasis on *Lactobacillus brevis* as a functional dairy starter. Crit. Rev. Food Sci. Nutr..

[10] Li H., Gao D., Cao Y., Xu H. (2008). A high γ-aminobutyric acid-producing *Lactobacillus brevis* isolated from Chinese traditional paocai. Ann. Microbiol..

[11] Li H., Qiu T., Huang G., Cao Y. (2010). Production of gamma-aminobutyric acid by *Lactobacillus brevis* NCL912 using fed-batch fermentation. Microb. Cell Factories.

[12] Li H., Qiu T., Cao Y., Yang J., Huang Z. (2009). Pre-staining paper chromatography method for quantification of γ-aminobutyric acid. J. Chromatogr. A.

[13] Qiu T., Li H., Cao Y. (2010). Pre-staining thin layer chromatography method for amino acid detection. Afr. J. Biotechnol..

[14] Tsukatani T., Higuchi T., Matsumoto K. (2005). Enzyme-based microtiter plate assay for γ-aminobutyric acid: Application to the screening of γ-aminobutyric acid producing lactic acid bacteria. Anal. Chim. Acta.

[15] Yao L., Cao J., Lyu C., Fan F., Wang H., Cao H., Huang J., Mei L. (2021). Food-grade γ-aminobutyric acid production by immobilized glutamate decarboxylase from *Lactobacillus plantarum* in rice vinegar and monosodium glutamate system. Biotechnol. Lett..

[16] Gao D., Chang K., Ding G., Wu H., Chen Y., Jia M., Liu X., Wang S., Jin Y., Pan H. (2019). Genomic insights into a robust gamma-aminobutyric acid producer *Lactobacillus brevis* CD0817. AMB Express.

[17] Lim H.J., Jung D.H., Cho E.S., Seo M.J. (2022). Expression, purification, and characterization of glutamate decarboxylase from human gut-originated *Lactococcus garvieae* MJF010. World J. Microbiol. Biotechnol..

[18] Mousavi R., Mottawea W., Hassan H., Gomaa A., Audet M.C., Hammami R. (2022). Screening, characterization and growth of γ-aminobutyric acid-producing probiotic candidates from food origin under simulated colonic conditions. J. Appl. Microbiol..

[19] Li H., Ding D., Cao Y., Yu B., Guo L., Liu X. (2015). Partially overlapping primer-based PCR for genome walking. PLoS ONE.

[20] Chang K., Wang Q., Shi X., Wang S., Wu H., Nie L., Li H. (2018). Stepwise partially overlapping primer-based PCR for genome walking. AMB Express.

[21] Wang L., Jia M., Li Z., Liu X., Sun T., Pei J., Wei C., Lin Z., Li H. (2022). Wristwatch PCR: A versatile and efficient genome walking strategy. Front. Bioeng. Biotechnol..

[22] Wang L., Jia M., Li Z., Liu X., Sun T., Pei J., Wei C., Lin Z., Li H. (2023). Protocol to access unknown flanking DNA sequences using wristwatch-PCR for genome-walking. STAR Protoc..

[23] Sun T., Jia M., Wang L., Li Z., Lin Z., Wei C., Pei J., Li H. (2022). DAR-PCR: A new tool for efficient retrieval of unknown flanking genomic DNA. AMB Express.

[24] Pei J., Sun T., Wang L., Pan Z., Guo X., Li H. (2022). Recombinant sequence-specific primer driven racket PCR: A simple and practical tool for genome walking. Front. Genet..

[25] Wei C., Lin Z., Pei J., Pan H., Li H. (2023). Semi-site-specific primer PCR: A simple but reliable genome-walking tool. Curr. Issues Mol. Biol..

[26] Lin Z., Wei C., Pei J., Li H. (2023). Bridging PCR: An efficient and reliable scheme implemented for genome-walking. Curr. Issues Mol. Biol..

[27] Kotik M. (2009). Novel genes retrieved from environmental DNA by polymerase chain reaction: Current genome-walking techniques for future metagenome applications. J. Biotechnol..

[28] Wu Q., Shah N.P. (2018). Restoration of GABA production machinery in *Lactobacillus brevis* by accessible carbohydrates, anaerobiosis and early acidification. Food Microbiol..

[29] Gong L., Ren C., Xu Y. (2020). *GlnR* negatively regulates glutamate-dependent acid resistance in *Lactobacillus brevis*. Appl. Environ. Microbiol..

[30] Lyu C., Yao L., Zhu Q., Mei J., Cao Y., Hu S., Zhao W., Huang J., Mei L., Yao S. (2021). Reconstruction of the glutamate decarboxylase system in *Lactococcus lactis* for biosynthesis of food-grade γ-aminobutyric acid. Appl. Microbiol. Biotechnol..

[31] Zhang Y., Song L., Gao Q., Yu S.M., Li L., Gao N.F. (2012). The two-step biotransformation of monosodium glutamate to GABA by *Lactobacillus brevis* growing and resting cells. Appl. Microbiol. Biotechnol..

[32] Binh T.T., Ju W.T., Jung W.J., Park R.D. (2014). Optimization of γ-amino butyric acid production in a newly isolated *Lactobacillus brevis*. Biotechnol. Lett..

[33] Zhao A., Hu X., Pan L., Wang X. (2015). Isolation and characterization of a gamma-aminobutyric acid producing strain *Lactobacillus buchneri* WPZ001 that could efficiently utilize xylose and corncob hydrolysate. Appl. Microbiol. Biotechnol..

[34] Li H., Qiu T., Chen Y., Cao Y. (2011). Separation of gamma-aminobutyric acid from fermented broth. J. Ind. Microbiol. Biotechnol..

[35] Li H., Qiu T., Liu X., Cao Y. (2013). Continuous cultivation of *Lactobacillus brevis* NCL912 for production of gamma-aminobutyric acid. Ann. Microbiol..

[36] Jia M., Zhu Y., Wang L., Sun T., Pan H., Li H. (2022). pH auto-sustain-based fermentation supports efficient gamma-aminobutyric acid production by *Lactobacillus brevis* CD0817. Fermentation.

[37] Wang Q., Liu X., Fu J., Wang S., Chen Y., Chang K., Li H. (2018). Substrate sustained release-based high efficacy biosynthesis of GABA by *Lactobacillus brevis* NCL912. Microb. Cell Factories.

[38] Li H., Qiu T., Gao D., Cao Y. (2010). Medium optimization for production of gamma-aminobutyric acid by *Lactobacillus brevis* NCL912. Amino Acids.

[39] Chen Y., Chang K., Xie X., Liu X., Jia M., Nie L., Li H., Wang S. (2019). Disassociation of glutamate from γ-aminobutyric acid by zinc acetate-assisted differential precipitation/dissolution: Application to the quantification of γ-aminobutyric acid. J. Chromatogr. A.

[40] Li H., Sun T., Jia M., Wang L., Wei C., Pei J., Lin Z., Wang S. (2022). Production of gamma-aminobutyric acid by *Levilactobacillus brevis* CD0817 by coupling fermentation with self-buffered whole-cell catalysis. Fermentation.

[41] Li H., Wang L., Nie L., Liu X., Fu J. (2023). Sensitivity intensified ninhydrin-based chromogenic system by ethanol-ethyl acetate: Application to relative quantitation of GABA. Metabolites.

[42] Wu C.H., Hsueh Y.H., Kuo J.M., Liu S.J. (2018). Characterization of a potential probiotic *Lactobacillus brevis* RK03 and efficient production of γ-aminobutyric acid in batch fermentation. Int. J. Mol. Sci..

[43] Seo M.J., Nam Y.D., Lee S.Y., Park S.L., Yi S.H., Lim S.I. (2013). Expression and characterization of a glutamate decarboxylase from *Lactobacillus brevis* 877G producing γ-aminobutyric acid. Biosci. Biotechnol. Biochem..

[44] Wu Q., Tun H.M., Law Y.S., Khafipour E., Shah N.P. (2017). Common distribution of *gad* operon in *Lactobacillus brevis* and its GadA contributes to efficient GABA synthesis toward cytosolic near-neutral pH. Front. Microbiol..

[45] Park S.J., Kim D.H., Kang H.J., Shin M., Yang S.Y., Yang J., Jung Y.H. (2021). Enhanced production of gamma-aminobutyric acid (GABA) using *Lactobacillus plantarum* EJ2014 with simple medium composition. LWT-Food Sci. Technol..

[46] Lim H.S., Cha I.T., Roh S.W., Shin H.H., Seo M.J. (2017). Enhanced production of gamma-aminobutyric acid by optimizing culture conditions of *Lactobacillus brevis* HYE1 isolated from kimchi, a Korean fermented food. J. Microbiol. Biotechnol..

[47] Alizadeh Behbahani B., Jooyandeh H., Falah F., Vasiee A. (2020). Gamma-aminobutyric acid production by *Lactobacillus brevis* A3: Optimization of production, antioxidant potential, cell toxicity, and antimicrobial activity. Food Sci. Nutr..

[48] Abbasiliasi S., Tan J.S., Ibrahim T.A.T., Bashokouh F., Ramakrishnan N.R., Mustafa S., Ariff A.B. (2017). Fermentation factors influencing the production of bacteriocins by lactic acid bacteria: A review. RSC Adv..

[49] Juarez del Valle M., Laiño J.E., Savoy de Giori G., LeBlanc J.G. (2017). Factors stimulating riboflavin produced by *Lactobacillus plantarum* CRL725 grown in a semi-defined medium. J. Basic Microbiol..

[50] Cataldo P.G., Villegas J.M., de Giori G.S., Saavedra L., Hebert E.M. (2020). Enhancement of γ-aminobutyric acid (GABA) production by *Lactobacillus brevis* CRL 2013 based on carbohydrate fermentation. Int. J. Food Microbiol..

[51] Tanamool V., Hongsachart P., Soemphol W. (2019). Screening and characterisation of gamma-aminobutyric acid (GABA) producing lactic acid bacteria isolated from Thai fermented fish (Plaa-som) in Nong Khai and its application in Thai fermented vegetables (Som-pak). Food Sci. Technol..

[52] Yang S., Lin Q., Lu Z., Lü F., Bie X., Zou X., Sun L. (2008). Characterization of a novel glutamate decarboxylase from streptococcus salivarius ssp. thermophilus Y2. J. Chem. Technol. Biotechnol..

[53] Villegas J.M., Brown L., de Giori G.S., Hebert E.M. (2016). Optimization of batch culture conditions for GABA production by *Lactobacillus brevis* CRL1942, isolated from quinoa sourdough. LWT-Food Sci. Technol..

[54] Bremer E., Krämer R. (2019). Responses of microorganisms to osmotic stress. Annu. Rev. Microbiol..

[55] Laroute V., Mazzoli R., Loubière P., Pessione E., Cocaign-Bousquet M. (2021). Environmental conditions affecting GABA production in *Lactococcus lactis* NCDO 2118. Microorganisms.

[56] Cotter P.D., Hill C. (2003). Surviving the acid test: Responses of gram-positive bacteria to low pH. Microbiol. Mol. Biol. Rev..

[57] Small P.L., Waterman S.R. (1998). Acid stress, anaerobiosis and *gadCB*: Lessons from *Lactococcus lactis* and *Escherichia coli*. Trends Microbiol..

[58] Lyu C., Zhao W., Peng C., Hu S., Fang H., Hua Y., Yao S., Huang J., Mei L. (2018). Exploring the contributions of two glutamate decarboxylase isozymes in *Lactobacillus brevis* to acid resistance and γ-aminobutyric acid production. Microb. Cell Factories.

[59] Higuchi T., Hayashi H., Abe K. (1997). Exchange of glutamate and gamma-aminobutyrate in a *Lactobacillus* strain. J. Bacteriol..

[60] Xiao T., Shah N.P. (2021). Lactic acid produced by streptococcus thermophilus activated glutamate decarboxylase (GadA) in *Lactobacillus brevis* NPS-QW 145 to improve γ-amino butyric acid production during soymilk fermentation. LWT-Food Sci. Technol..

